# Head and pelvic movement asymmetries at trot in riding horses in training and perceived as free from lameness by the owner

**DOI:** 10.1371/journal.pone.0176253

**Published:** 2017-04-25

**Authors:** Marie Rhodin, Agneta Egenvall, Pia Haubro Andersen, Thilo Pfau

**Affiliations:** 1 Department of Clinical Sciences, Swedish University of Agricultural Sciences, Uppsala, Sweden; 2 Department of Clinical Science and Services, The Royal Veterinary College, University of London, Hawkshead Lane, North Mymms, Hatfield, United Kingdom; University of Illinois, UNITED STATES

## Abstract

Recent studies evaluating horses in training and considered free from lameness by their owners have identified a large proportion of horses with motion asymmetries. However the prevalence, type and magnitude of asymmetries when trotting in a straight line or on the lunge have not been investigated. The aim of this study was to objectively investigate the presence of motion asymmetries in riding horses in training by identifying the side and quantifying the degree and type (impact, pushoff) of forelimb and hind limb asymmetries found during straight line trot and on the lunge. In a cross-sectional study, vertical head and pelvic movement symmetry was measured in 222 Warmblood type riding horses, all without perceived performance issues and considered free from lameness by their owners. Body-mounted uni-axial accelerometers were used and differences between maximum and minimum head (HDmax, HDmin) and pelvic (PDmax, PDmin) vertical displacement between left and right forelimb and hind limb stances were calculated during straight line trot and on the lunge. Previously reported symmetry thresholds were used. The thresholds for symmetry were exceeded in 161 horses for at least one variable while trotting in a straight line, HDmin (n = 58, mean 14.3 mm, SD 7.1), HDmax (n = 41, mean 12.7 mm, SD 5.5), PDmax (n = 87, mean 6.5 mm, SD 3.10), PDmin (n = 79, mean 5.7 mm, SD 2.1). Contralateral and ipsilateral concurrent forelimb and hind limb asymmetries were detected in 41 and 49 horses, respectively. There was a linear association between the straight line PDmin values and the values on the lunge with the lame limb to the inside of the circle. A large proportion (72.5%) of horses in training which were perceived as free from lameness by their owner showed movement asymmetries above previously reported asymmetry thresholds during straight line trot. It is not known to what extent these asymmetries are related to pain or to mechanical abnormalities. Therefore, one of the most important questions that must be addressed is how objective asymmetry scores can be translated into pain, orthopedic abnormality, or any type of unsoundness.

## Introduction

Orthopedic diseases constitute the most common group of health problems in riding horses and remain one of the most common causes of interrupting the athletic careers of horses [1,2]. The median length of life for geldings and stallions in Swedish Warmblood horses is 15 years [3], the age at which many horses can perform at the highest level if they are healthy. The clinical lameness examination has been the prime tool to detect and investigate orthopedic diseases for decades, but currently applied clinical methods appear rather insensitive to subtle pain and pathology with resulting small changes in motion symmetry [4–6]. Diseases causing subtle lameness may hence persist and develop into chronicity before overt clinical signs of lameness are developed. The weaknesses of visual assessment in horses with mild to moderate lameness have been described in numerous studies and comprise low inter-rater agreement among clinicians, expectation bias and inaccuracy [4–7]. Therefore early, precise and accurate recognition of lameness with biomechanical methods has been the focus of research in recent decades. In trot, the non-lame horse shows a symmetric sinusoidal motion pattern of head and pelvis which undergoes systematic changes when loading of the limbs changes, for example as a result of lameness [8–10]. The differences in maximum and minimum position of head or pelvis between left/right stances (HDmin, HDmax, PDmin and PDmax) are examples of symmetry measures commonly used for quantification of lameness and are directly linked to the underlying changes in limb loading and propulsion [10,11]. Today, commercially available biomechanical techniques are being developed and validated for detecting changes in gait symmetry suitable for research and clinical practice [12–14]. However, only few studies have correlated degree of clinical lameness to the biomechanical results of symmetry measurements. It is essential to improve our knowledge on how objectively measured asymmetry scores can be translated into pain, dysfunction, orthopedic abnormalities, or any type of unsoundness.

Many so called “owner-sound” horses, free from lameness according to the judgement of their owner, that are in regular training and competition show asymmetric motion patterns to a similar degree as horses examined and treated for orthopedic pathology. Two studies, objectively evaluating the motion pattern of 201 and 23 riding horses in training and judged as sound by their owners, showed that 107 (53%) and 14 (61%) horses, respectively, had asymmetric head and/or pelvic movements measured during straight line trot [15,16]. In two studies [17,18] 506 and 57 “owner-sound” horses were evaluated by visual assessment of the motion pattern under different circumstances (in straight line trot, lungeing and when ridden) and 46% and 65% horses respectively showed abnormal movements interpreted as pathological conditions. Landman et al. [19] found that 19.5% of 399 horses examined before purchase showed a lameness of degree two or more on a 0–5 scale. None of these studies quantified the distribution of forelimb or hind limb lameness, the degree of asymmetry or how the asymmetric motion pattern was affected by circular motion, i.e. when trotting on the lunge, a commonly employed exercise during lameness and pre-purchase examinations. It is not known whether these asymmetries are influenced by horse age, horse size, equestrian discipline or the horse’s performance level. Murray et al. [20] investigated the association of type of sport and performance level with specific orthopedic conditions and found that show jumping horses were at increased risk of forelimb deep digital flexor tendinitis whereas dressage horses were at significantly increased risk of hind limb suspensory ligament injury. Whether these differences between disciplines may also be reflected by differences in motion symmetry among horses in training has never been investigated.

A recent study of 60 performing polo ponies revealed that 60–67% showed gait asymmetries which were not related to age or preferentially left or right sided [21] but the clinical relevance of these asymmetries are still unknown.

Most studies on lameness have been performed in horses trotting on the straight. However lungeing is commonly used during lameness and pre-purchase evaluations since it induces differences in the severity of apparent lameness compared to trot on the straight [22]. Lungeing also affects the symmetry of the motion pattern in sound horses [23,24] and this circle-dependent asymmetry will be superimposed on the pre-existing lameness of the horse making it more, or less, visible [23]. Whether this circle-dependent asymmetry will increase or decrease the degree of asymmetry depends on whether the limb contributing to the asymmetry is on the inside or outside of the circle [23]. This phenomenon is very relevant for diagnostic procedures in clinical cases but has not yet been investigated thoroughly.

The degree of lameness varies between strides but how this inter-stride variability changes with increased degree of lameness is not well described and may be important knowledge when evaluating lame horses [25]. It is also not described for different lameness scales whether the scoring should be based on the lamest stride or an average of all strides during a trot up or which strides should be used for comparison before and after diagnostic analgesia [26].

The aim of this study was to objectively investigate prevalence and nature of motion asymmetries in riding horses in training, perceived as being free from lameness by their rider, by identifying the side and quantifying the degree and type (impact, pushoff) of forelimb and hind limb asymmetries during trot on the straight and on the lunge, using a commercial sensor-based system for lameness detection. We hypothesised that the effect of lungeing would neutralise the asymmetry seen on straight line trot in one lungeing direction and exacerbate the asymmetry in the opposite direction. We also hypothesized that stride variability during a test run decreases with increased degree of asymmetry and therefore the association between proportion of left /right asymmetric strides and degree of asymmetry was investigated. Finally, we wanted to investigate if discipline, performance level, age, gender and size of the horse were related to the prevalence of gait asymmetry.

## Material and methods

This study has been approved by the Ethical Committee for Animal Experiments, Uppsala, Sweden (permission number C251/9, C48/13) and informed consent for data collection was obtained from the horse owners prior to the study.

### Study design

Cross-sectional study of movement symmetry in riding horses in training.

### Horses

Invitations, containing an informed consent form, to participate in the study were extended to horse owners based on convenience sampling between 2009 and 2014. Inclusion criteria: Horses (>148 cm height) perceived to be free from lameness by their owner/rider (interview) and ridden regularly, at least 2–3 times per week. Exclusion criteria were reports of lameness, health problems or medical treatments within the last 6 months or horses not cooperating when ridden or on the lunge. Gender, age, breed, height, discipline and level of performance were recorded for each horse according to information given by the owner. There were 81 mares, 3 stallions and 138 geldings with an age distribution of 3–25 years (median 10 years) and a withers height of 150–182 cm (median 165 cm). The horses belonged to 122 different owners (range 1–58 horses/owner, median 1 horse/owner). The three owners with the highest number of horses (58, 10 and 8 horses) were teaching facilities where the horses were ridden by different riders. Of the 222 horses, there were 219 Warmblood type horses and 3 crossbreeds of unknown breed.

### Data collection protocol

Motion analysis of the horses in trot, when handled by the owner, was performed in three trials in random order: in a straight (S) line on a hard surface (concrete or gravel based); during lungeing on a soft surface, (sand or fibre based) to the left (L); and during lungeing to the right (R). Circle diameters varied between 10–15 m, using similar diameters within horse for the left and right reins.

## Motion analysis

A commercial inertial measure unit (IMU) based gait analysis system (Lameness Locator, Equinosis, St. Louis, MO, USA) for lameness detection was used [14]. Briefly, one uni-axial accelerometer was mounted to a head bumper attached to the bridle over the poll and one uni-axial accelerometer was taped to the midline of the pelvis at the level of the tubera sacrale. Finally, one uni-axial gyroscope was attached dorsally to the proximal and middle phalanges of the right forelimb. Vertical uni-axial acceleration was recorded at 200 Hz with 8 bit digital resolution and data were transmitted wirelessly from the sensors to a nearby laptop computer running the data collection software.

### Data processing

**Fig 1:**
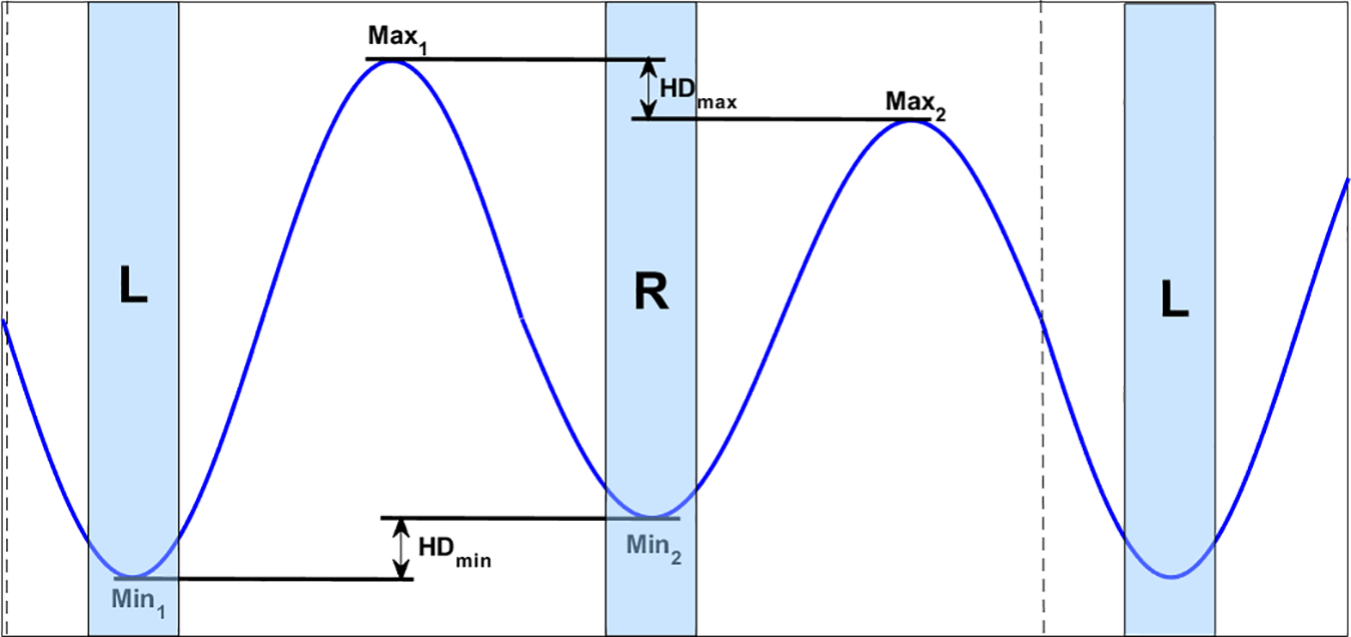
Example of vertical head movement in a horse with right forelimb lameness resulting in positive HDmin and HDmax values. Approximate timing of left (L) and right (R) forelimb stance is indicated by grey bars. Pelvic movement symmetry is calculated in the same way from vertical pelvis movement signal.

Data from all straight line and lungeing trials were processed with the software package for the gait analysis system. Raw uni-axial acceleration signals from head and pelvis sensors, aligned with the global vertical axis in the standing position, were first transformed into displacement signals using a custom-designed, error-correcting, double-integration technique and the signal from the right forelimb gyroscope was used for stride splitting [14]. From the displacement signal local maxima and minima were identified (two per stride). Differences in minimum head (HDmin) and pelvis (PDmin) height during left and right stance phases and differences in maximum head (HDmax) and pelvis (PDmax) height after left and right stance phases were computed for each stride (Fig 1).

Asymmetric head movement is attributed to forelimb asymmetry and asymmetric pelvis motion is attributed to hind limb asymmetry. Here, a horse presenting with reduced head movement during left front stance is referred to as left front asymmetric, a horse presenting with reduced pelvic movement during left hind stance as left hind asymmetric [9]. Removal of single outliers for the head movement was performed in the software package. The mean amplitude and sign of HDmin, HDmax, PDmin, and PDmax for all strides in a trial were calculated from the signal, resulting in either a positive or negative mean value for each variable and trial. Positive values indicated asymmetries attributed to the right and negative values asymmetries attributed to the left forelimb or hind limb HDmin_right/left_, HDmax_right/left_, PDmin_right/left_, and PDmax_right/left_. [9]. The number of left and right asymmetric strides according to the sign of HDmin and PDmin respectively were also calculated for each trial.

Criteria for motion asymmetry developed by Keegan et al. [14] were applied to absolute mean differences, over all strides in each horse’s straight line trial(s). Gait parameters of straight line and lungeing trials were retained for further analysis for horses with straight line values larger than 6 mm for the head movement (HDmin or HDmax) and 3 mm for the hind quarter (pelvic) motion (PDmin or PDmax) and with standard deviations less than their respective means.

For the horses with mean HDmax and/or HDmin values outside the thresholds the vector sum (VS) of the mean HDmax and HDmin was calculated as $\sqrt{{HDmax}^{2}+{HDmin}^{2}}$ and assigned to the left or right side (VS_left_ and VS_right_) depending on whether HDmin was positive or negative [9].

### Statistical methods

Descriptive statistics were calculated. To identify any relationships between motion asymmetry on the straight and on the lunge, the values for HDmin, HDmax, PDmin and PDmax during straight-line trot were plotted (on the x-axis, for visual evidence of linearity) and regressed against HDmin, HDmax, PDmin and PDmax values during lungeing (y-axis). This procedure was implemented three times: 1) for trials with the asymmetric limb to the inside of the circle, 2) with the asymmetric limb to the outside of the circle and also against 3) the sum of the excursion values from both lungeing directions. Likewise the HDmin and PDmin values during straight line trot were regressed against the proportion of right forelimb and right hind limb asymmetric strides, respectively, to identify any relationship between the degree of movement asymmetry on the straight (only straight) and the proportion of left/right asymmetric strides.

Mixed model analysis (SAS Institute Inc. (2014) SAS/STAT 13.2 User’s Guide, SAS Institute Inc, Cary, North Carolina) was performed for the analyses with respect to gender, age, height and discipline, all included as fixed effects, and absolute values of the outcome variables (HDmin, HDmax, PDmin and PDmax) were used. These outcome variables were not normally distributed. Box-Cox transformation along Tukeys ladder of powers was performed and used to select the best transformation closest to normality. Each of the independent variables was tried as a fixed effect one at a time, while controlling for direction as a fixed effect. The format of the tested variables was as follows: male horses (geldings and stallions) were compared with mares; main discipline of the horses was divided into general (including 5 event horses), show-jumping or dressage. Further, height at the withers was divided into four categories [<155 cm, 155<164 cm, 164<172 cm and >172 cm] and age into 4 categories [<6 years, 6<11 years, 11<16 years and >16 years]. These dummy variables suggested that height at withers and age were reasonably linear when modelled against the outcomes and were therefore modelled as linear. Random effect was horse within owner. The covariance structure was variance component. Models are shown if group p-values <0.05 (type III) and at least one pairwise comparison p <0.05. A description of the variables used and the data is available in supporting information (S1 Data).

## Results

### Study population

Of the 222 horses, 161 (72.5%) Warmblood type horses had absolute values outside thresholds for straight line trot for at least one symmetry variable (HDmin (n = 58, mean 14.3, median 11.8, range 6.6–35.2 mm), HDmax (n = 41, mean 12.7, median 11.1, range 6.4–35.3 mm), PDmin (n = 79, mean 5.6, median 5.1, range 3.1–12.3 mm) and PDmax (n = 87, mean 6.5, median 5.4, range 3.1–15.4 mm). These horses were retained for further analysis and the horses with symmetrical movement on the straight were excluded.

Of the 161 horses, 57 were mares, 3 were stallions and 101 were geldings with an age distribution of 3–25 years (median 11 years), withers height 150–182 cm (median 165 cm, data on height was missing in 19 horses) and the horses belonged to 74 different owners (range 1–44 horses/owner, median 1 horse/owner).

For straight line trot there were (mean ± SD), 30 ±12.3 strides evaluated per trial. For lungeing to the left and right 47 ±16.7 respectively 49 ±17.4 strides were evaluated per trial. Contralateral and ipsilateral concurrent forelimb and hind limb asymmetries on the straight were detected in 41 and 49 horses, respectively.

### Effect of lungeing direction

Mean HDmin_right_, HDmin_left_, HDmax_right_, HDmax_left_, VS_right_, VS_left_, PDmin_right_, PDmin_left_, PDmax_right_ and PDmax_left_ values for all horses exceeding the thresholds for straight line trot and their values on the lunge are shown in Table 1.

**Table 1:** Forelimb (HDmin, HDmax and VS) and hind limb (PDmin and PDmax) asymmetries, are presented for the horses (n) exceeding the threshold value for symmetry for straight line motion and their corresponding values for the left and right lungeing direction. Positive values are attributed to a right limb and negative values a left limb asymmetry.

				Percentiles				
Variable	n	Mean	Std	5th	25th	50th	75th	95th
**Straight**								
HDmin_right_	29	12.8	6.4	7.2	8.7	11.1	15.2	23.9
HDmin_left_	29	-15.8	7.5	-30.5	-20.2	-13.5	-10.0	-7.9
HDmax_right_	24	11.3	3.6	7.4	8.5	10.7	13.5	17.4
HDmax_left_	17	-14.6	7.2	-35.3	-17.4	-13.5	-9.3	-6.4
VSleft	40	17.4	8.3	9.0	11.8	15.5	20.9	35.0
Vsright	40	14.1	5.9	7.2	9.7	13.0	16.0	24.7
PDmin_right_	36	5.8	2.3	3.5	4.2	5.0	7.2	11.1
PDmin_left_	43	-5.5	1.8	-8.7	-6.1	-5.2	-3.9	-3.4
PDmax_right_	43	6.9	3.4	3.5	4.1	5.4	9.6	13.6
PDmax_left_	44	-6.1	2.8	-12.5	-6.6	-5.3	-4.3	-3.7
**Left circle**								
HDmin_right_	29	11.0	12.6	-3.7	1.5	9.7	15.4	34.8
HDmin_left_	29	-7.6	14.2	-35.5	-14.0	-10.4	-4.4	17.4
HDmax_right_	24	4.0	9.0	-8.9	-2.7	4.0	10.0	20.7
HDmax_left_	17	-3.6	11.3	-26.4	-12.1	-5.3	5.6	14.8
VSleft	40	15.9	9.9	3.7	10.3	12.8	20.1	36.9
VSright	40	16.0	10.7	2.6	9.3	13.9	20.5	38.9
PDmin_right_	36	-2.1	6.1	-11.7	-5.9	-1.0	0.8	5.1
PDmin_left_	43	-12.9	8.1	-24.2	-16.3	-12.7	-6.5	-3.7
PDmax_right_	43	5.9	6.3	-6.0	2.3	5.3	9.7	16.9
PDmax_left_	44	-1.9	4.0	-7.7	-4.1	-2.5	-0.1	4.9
**Right circle**								
HDmin_right_	29	2.1	14.4	-26.7	-6.5	14.0	10.5	25.2
HDmin_left_	29	-15.1	15.6	-40.7	-27.0	-12.0	-2.9	6.2
HDmax_right_	24	10.1	9.5	-4.1	3.2	8.9	18.0	24.7
HDmax_left_	17	-8.3	13.3	-35.5	-16.4	-4.8	-0.5	15.1
VSleft	40	21.1	14.0	3.1	10.2	18.8	27.3	52.0
VSright	40	16.2	11.6	4.7	7.9	12.8	19.0	37.5
PDmin_right_	36	12.5	6.9	-0.5	9.1	14.0	16.5	22.7
PDmin_left_	43	3.1	6.6	-4.8	-1.2	3.3	7.3	10.4
PDmax_right_	43	2.7	4.8	-4.6	-0.1	1.9	5.4	11.4
PDmax_left_	44	-7.0	4.9	-16.9	-9.7	-6.8	-3.6	-0.5

### Effect of age, level, discipline, gender and withers height

Outcome variables (absolute values of asymmetry variables) were modelled raised to the power of 0.25. PDmin was significantly (p = 0.01) higher for dressage horses (n = 65) (back-transformed value 5.3 mm) compared to show jumping horses (n = 33) (3.8 mm). In addition, controlling for direction, PDmin was also significantly (p = 0.001) higher on the left (5.9 mm) or right (5.9 mm) reins compared to straight line (2.7 mm) measurements.

PDmax was significantly higher (p = 0.01) for horses of advanced level (n = 12) (5.0 mm) compared to horses of intermediate level (n = 52) (3.1 mm). Models with a group p-value <0.05 are presented (S1 Table).

After controlling for owner, HDmin and HDmax were not affected by age, level, discipline, gender or withers height.

### Inter-stride variability

**Fig 2:**
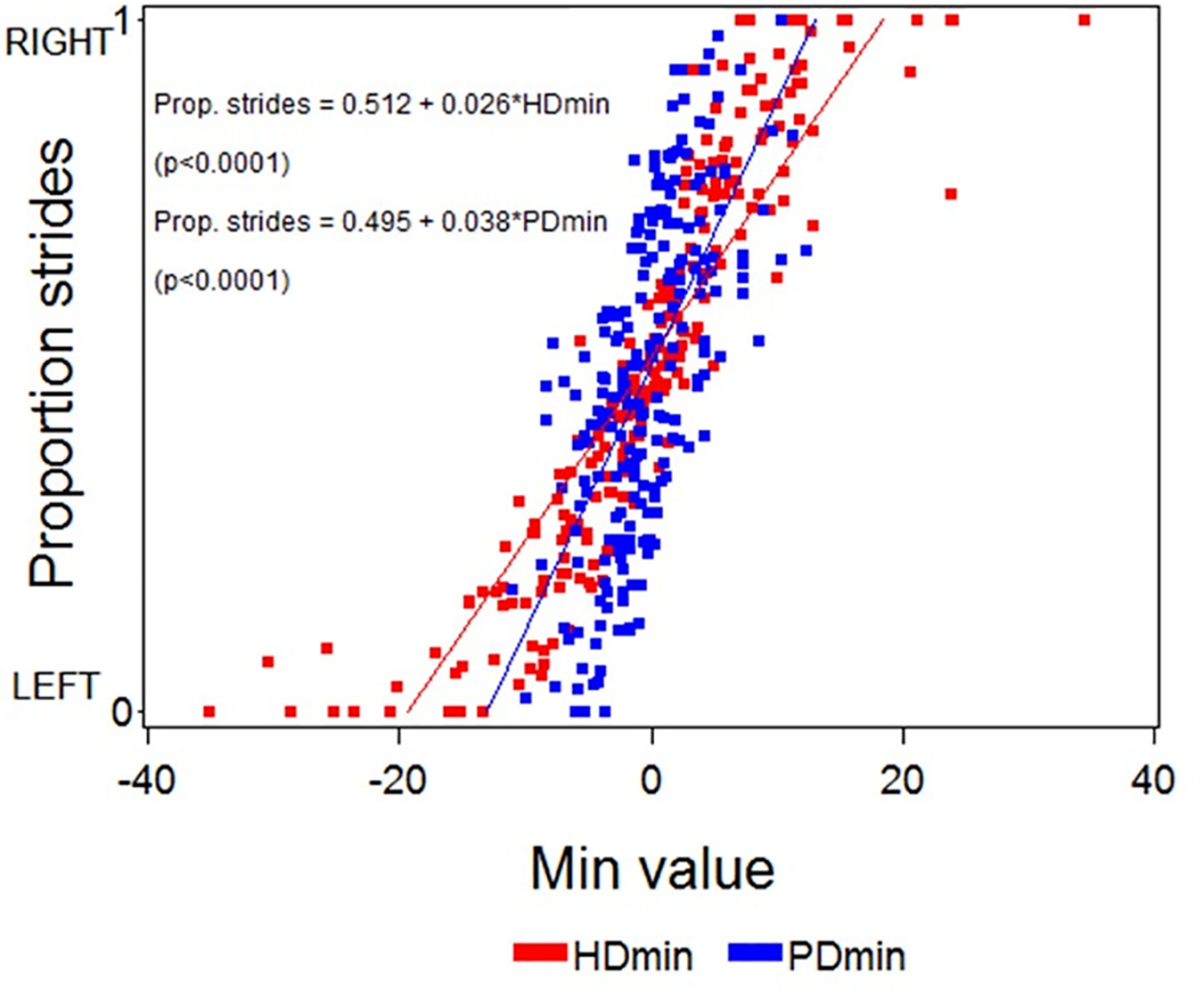
Scatter plot and regression analysis between mean values of differences in minimum head (HDmin) and pelvis (PDmin) position and the proportion of left/right limb asymmetric strides at straight-line trot for each trial. The p-values shown in the regression equations refers to the slopes (n = 161).

Scatter plots and regression lines/equations between the HDmin and PDmin values at straight line and the proportion of left or right limb asymmetric strides for all 161 horses are shown in Fig 2. Linear relationships between the mean value of HDmin and PDmin and the proportion of lame strides are demonstrated.

### Straight line asymmetries compared to asymmetries on the lunge

**Fig 3:**
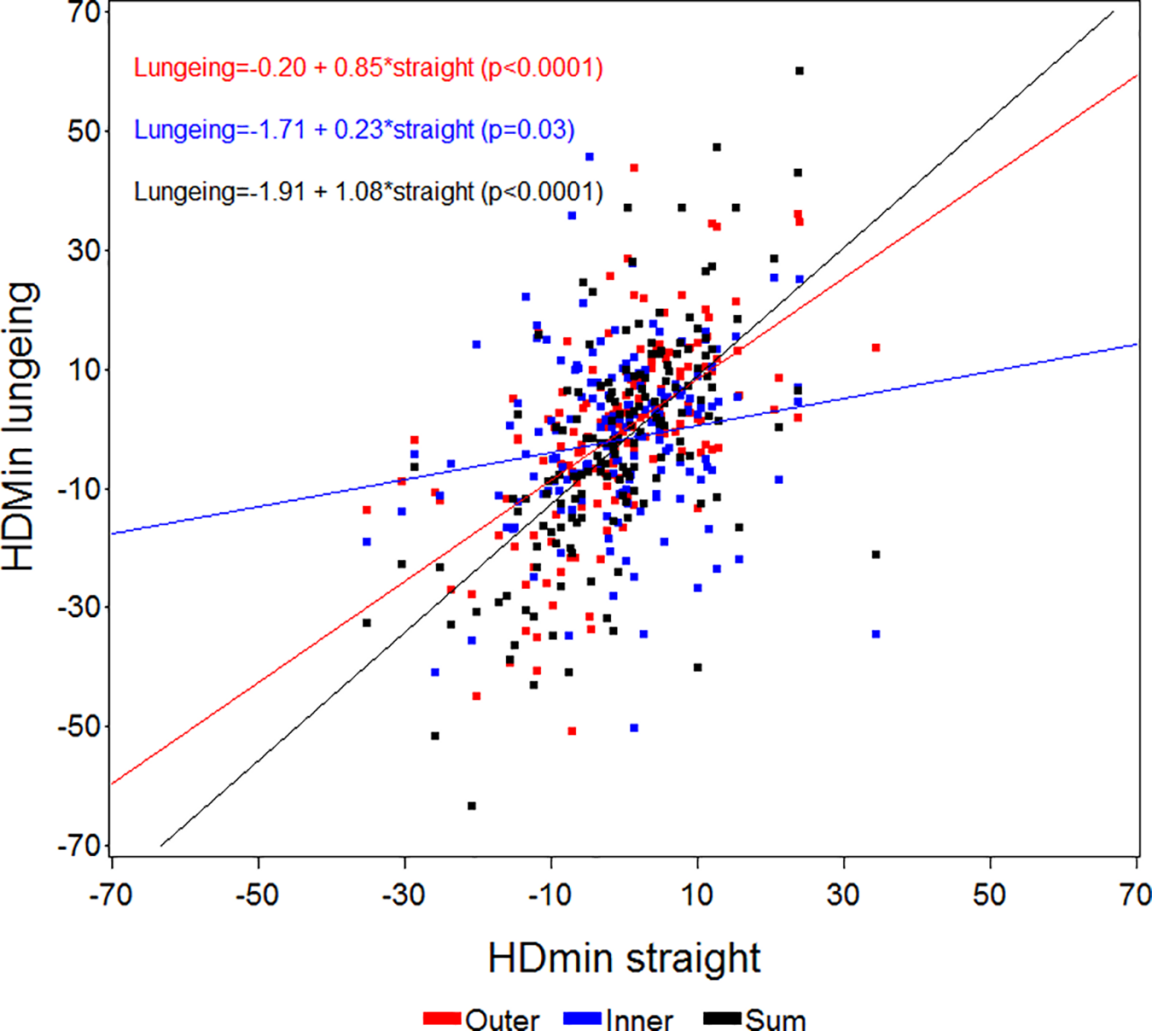
Scatter plot and regression analysis between straight line HDmin (mean values of differences in minimum head position) values (x-axis) and values from measurements on the lunge (y-axis) with the asymmetric limb to the inside (Inner) or outside (Outer) of the circle, or the sum (Sum) of the values from both lungeing directions for all horses (n = 161). The p-values shown refer to the slopes of the regression equations.

**Fig 4:**
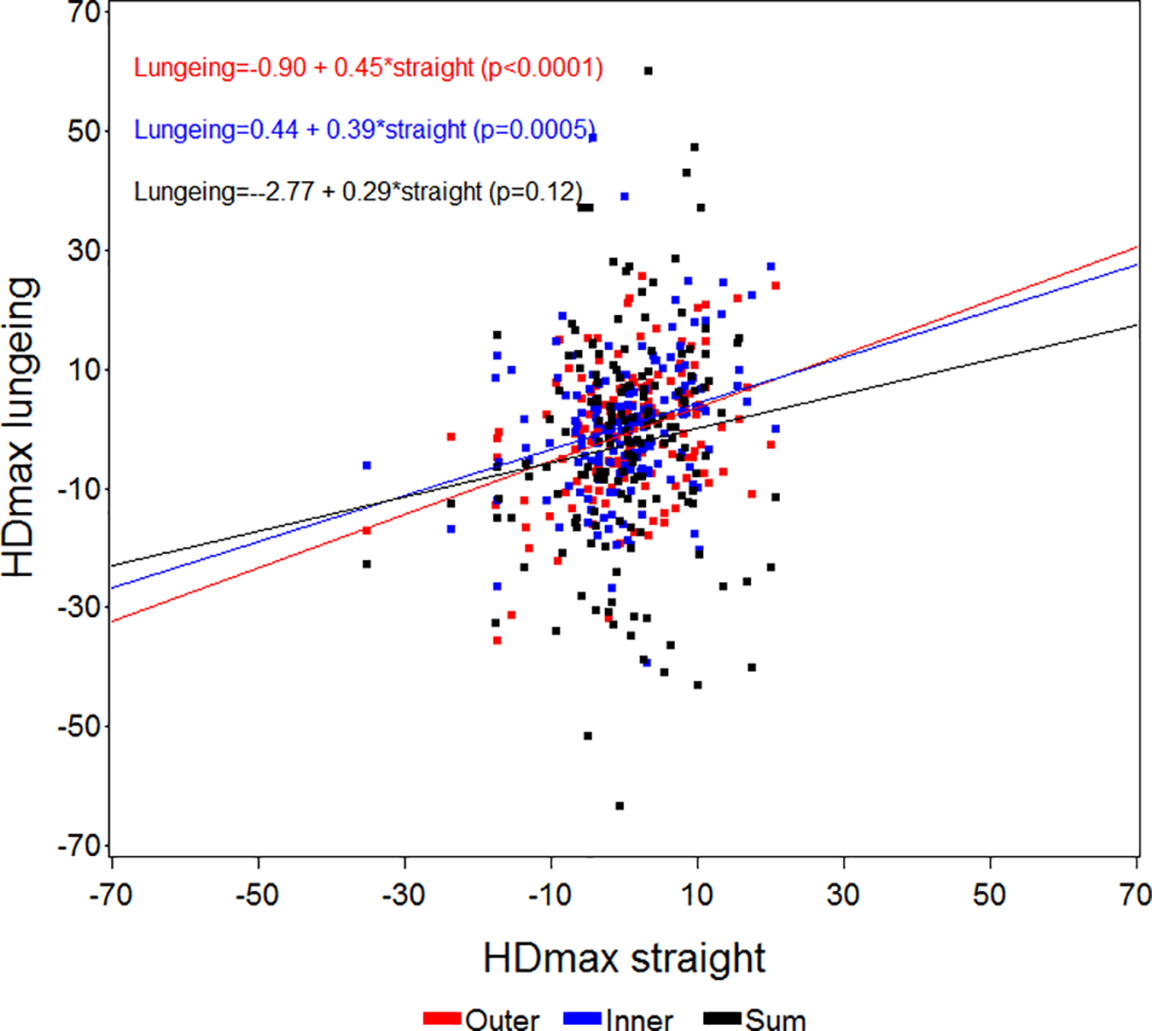
Scatter plot and regression analysis between straight line HDmax (mean values of differences in maximum head position) values (x-axis) and values from measurements on the lunge (y-axis) with the asymmetric limb to the inside (Inner) or outside (Outer) of the circle, or the sum (Sum) of the values from both lungeing directions for all horses (n = 161). The p-values shown refer to the slopes of the regression equations.

**Fig 5:**
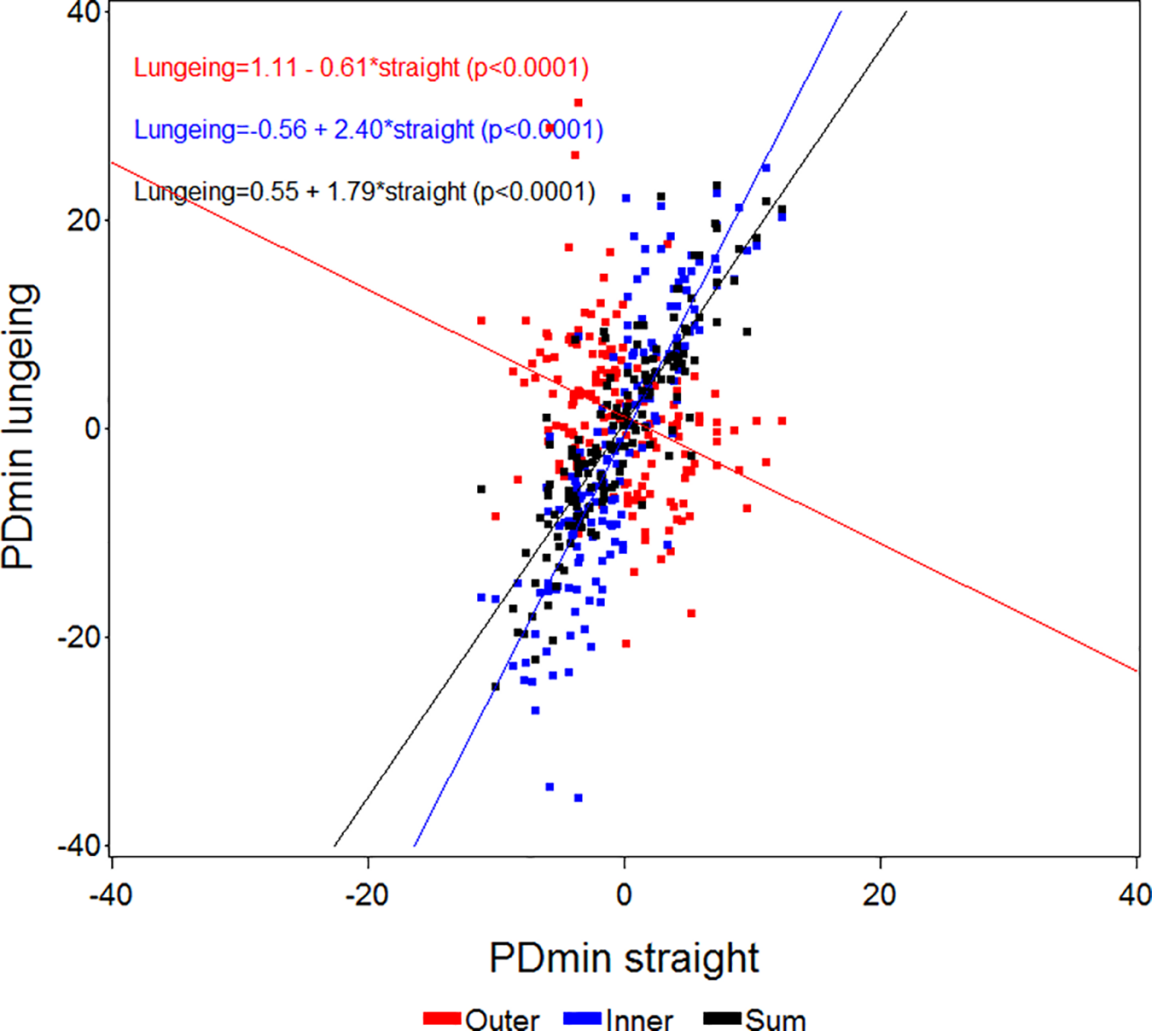
Scatter plot and regression analysis between straight line PDmin (mean values of differences in minimum pelvic position) values (x-axis) and values from measurements on the lunge (y-axis) with the asymmetric limb to the inside (Inner) or outside (Outer) of the circle, or the sum (Sum) of the values from both lungeing directions for all horses (n = 161). The p-values shown refer to the slopes of the regression equations.

**Fig 6:**
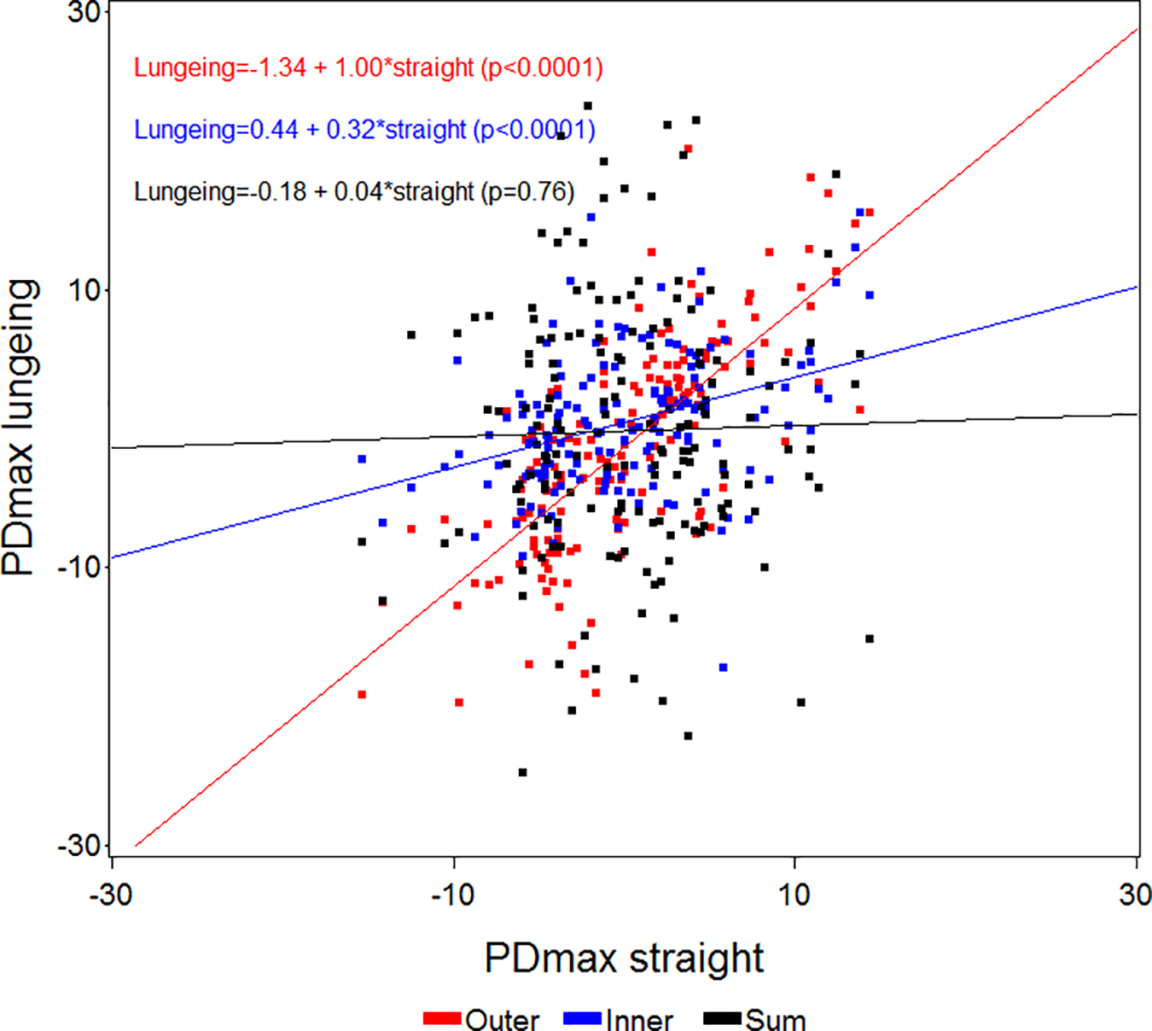
Scatter plot and regression analysis between straight line PDmax (mean values of differences in maximum head position) values (x-axis) and values from measurements on the lunge (y-axis) with the asymmetric limb to the inside (Inner) or outside (Outer) of the circle, or the sum (Sum) of the values from both lungeing directions for all horses (n = 161). The p-values shown refer to the slopes of the regression equations.

Figs 3–6 present scatter plots and regression lines/equations for the 161 horses. Comparisons were made for HDmin, HDmax, PDmin, PDmax values between straight line measurements and measurements on the lunge with the asymmetric limb to the inside (Inner) or outside (Outer) of the circle, or the sum (Sum) of the values from both lungeing directions. All regression lines showed a positive association between straight line values and either Inner, Outer or Sum except for the straight line PDmin values and the association to the Outer PDmin values, when the asymmetric limb was to the outside of the circle (Fig 5). All associations were significant except for the association between straight line HDmax and PDmax values to the values of the sum of both lungeing directions (Figs 4 and 6).

## Discussion

### Degree of asymmetries

In the present study, a remarkably high number of horses (72.5%) in training, judged as being free from lameness by their owner, show motion asymmetries. This raises the question whether these asymmetries are caused by pain and/or pathology or could simply be a biological variation.

In a previous study of ten horses with induced forelimb lameness [23], the mean degree of asymmetry for HDmin_left/right_ was -26.1/20.1 mm. The 5^th^ and 95^th^ percentiles of the present study exceed these values, presented in Table 1, but the mean values were lower (HDmin_left/right_ -15.8/12.8 mm). Further, the 10 horses with induced lameness showed HDmax_left/right_ values (-15.3/14.3 mm) of a similar degree to or slightly higher than the mean values of HDmax_left/right_ (-14.6/11.3 mm) for the horses in the present study. Maliye et al., [27,28] investigated 28 and 37 horses with naturally occurring forelimb and hind limb lameness respectively, evaluated both visually and objectively with the same system as in the present study, and obtained mean values of 18.3 mm (inter quartile range 12.2–32.0) for the VS (of HDmin and HDmax), compared to 16.8 mm in the present study for the forelimb asymmetric horses. For the clinically hind limb lame horses, the absolute mean PDmax was 5.10 mm (inter quartile range 2.95 to 8.77) compared to 6.9 mm for right limb and 6.1 mm for left asymmetric horses in the present study. These comparisons show that many horses, free from lameness according to their owners, present with objectively measured lameness parameters of the same magnitude or larger than horses thought to have clinically important lameness.

For the horses with induced hind limb lameness [23] the mean PDmin_left/right_ values were -9.0/6.2 mm and this degree is in accordance with six clinically hind limb lame horses investigated by Maliye et al., [28] with a group-mean value for PDmin of 6.0 mm (absolute value). The mean values for PDmin_left/right_ (-5.5/5.8 mm) were slightly lower in the present study but the 25^th^ percentile (-8.7) and 75^th^ percentile (7.2) exceeded these values. Thus, the results of the present study compared to similar results of previous studies of lameness in clinically lame horses using the same measuring system, indicate that there is considerable overlap between clinically lame horses and horses in training perceived as free from lameness according to their owner. For many horses in the current study, this means that, were they presented to a veterinarian with a complaint of poor performance or lameness (for whatever reason), the veterinarian would likely conduct a full lameness examination.

Asymmetries are generally caused by differences in loading and force production between limbs [11,29]. However, we do not know whether such asymmetries always are related to pain. This also means, that there is a limited understanding of to what extent asymmetries are related to an underlying disease process and whether their presence always poses a welfare issue. Only full clinical lameness examinations with diagnostic analgesia or analgesic testing by systemically administered analgesic drugs could possibly answer this question. Longitudinal studies on motion asymmetry and incidences of different orthopedic disorders are needed to answer this and other significant questions, as for example whether the higher pelvic motion asymmetries (PDmin) observed in the dressage horses in the present study indeed are related to an increased risk for developing hind limb suspensory ligament injuries that has been described by Murray et al. [20].

It may be speculated why owners and riders do not notice these asymmetries or which factors influence the decision of referring their horse to a veterinarian. One reason for not noticing that the horse moves asymmetrically could be that the horse is ridden on a soft surface, while asymmetry was measured on a hard surface, where disease processes might get provoked by increased forces, resulting in increased pain [16]. A common reason for referring a horse for a lameness examination is not only lameness, but also reduced performance or resistant behaviour of the horse when ridden, perhaps occurring with increasing frequency [22]. It may therefore also be possible that there was a difference in temperament between the horses investigated in the present study and horses examined for lameness.

Also, the riding skills and style of the rider may influence the symmetry of head and pelvic motion of the horse during riding and thus contribute to masking of forelimb asymmetry [29] or hind limb lameness during rising trot [30,31]. Riders or trainers may also interpret persistent movement asymmetries as laterality rather than signs of pain. We have no information about whether the horse’s movement had been evaluated regularly by rider, trainer or veterinarian. Based on our results, we recommend that the movement of horses is inspected regularly to facilitate detection of asymmetries at an early stage. Further studies need to be conducted to see whether these are linked to orthopedic problems. One recent study suggested that visual assessment of “saddle slip” i.e. saddle movement to one side during ridden exercise, could be a sign of hind limb lameness in ridden horses [17] that could be more readily detected by the rider or the trainer than a small asymmetric pelvic motion. Further studies are needed to verify this statement. The availability of smartphones and sensors that can measure and interpret acceleration data from horse movement suggests that owners may soon be able to track their horse’s movement through time, which opens up new possibilities for early lameness detection [32]. Adequate methods of training horse owners to undertake this task with the necessary repeatability and to develop automated methods for assessing data quality should be investigated. The emergence of these technologies warrants the continued investigation of the biological meaning of movement asymmetries.

### Lungeing

The strong effects of lungeing on the symmetry of the horses in this study confirms that circle dependent effects influence motion symmetry in both horses free from lameness and horses with induced lameness [15,23]. In the present study when lunged on a soft surface, horses with PDmin values outside normal limits on the straight line showed increased asymmetry when the asymmetric limb was on the inside of the circle and decreased asymmetry when the limb was on the outside of the circle (Table 1). The opposite was seen for PDmax asymmetries. Horses with HDmin values outside normal limits were more asymmetric with the asymmetric limb on the outside of the circle and showed more symmetrical movement with the asymmetric limb on the inside of the circle. The circle-dependent findings for head movement were less consistent than for pelvic motion and some horses switched the side of the asymmetry (Table 1) during lungeing compared to straight line measurement, showing bilateral asymmetries.

Since circular movement induces asymmetries, especially in the pelvis (PDmin), the initial asymmetry observed on the straight may be masked with the asymmetric limb on the outside of the circle (Fig 5). Therefore when evaluating horses on the lunge, both an increase or a decrease in symmetry could be a sign of positive diagnostic analgesia or of effective treatment depending on the lame limb being on the inside or the outside of the circle. The high correlation between straight line asymmetries and the sum of both lungeing directions indicates that the asymmetries generally are amplified by the circle dependent asymmetries in one direction and attenuated when changing direction. This is also in accordance with empiric clinical experience. We believe that lungeing simply increases the degree of asymmetry from being difficult or impossible to detect by eye to more clearly exceeding the previously investigated limits for human visual perception [33]. The benefits of lungeing may hence be more unclear, if the lameness is readily visible (or at least measurable) on the straight line. Lungeing might increase the accuracy of detecting the primary lame limb but could also decrease the accuracy due to the low inter-rater agreement when assessing lameness on the lunge [4]. In clinical text books, descriptions of increased lameness for certain orthopedic disorders are given when the affected limb is on the outside (proximal suspensory desmitis, medial carpal problems, medial sesamoid bone problems) or on the inside (bilateral forelimb lameness) of the circle [22]. Quantitative assessment may provide essential evidence for validating these observations through application of diagnostic analgesia to localize the origin of pain within the affected limb. Such important methodological questions can be answered by biomechanical studies of large populations of well documented clinical cases, before and after diagnostic analgesia. In the present study 49 horses showed ipsilateral and 41 horses showed contralateral concurrent forelimb and hind limb asymmetries and we do not know if these asymmetry patterns reflect single or multi limb lameness. There may also be differences in terms of the measured asymmetries during circular movement with different effects on primary and compensatory movement asymmetry. This needs further investigation ideally in horses with diagnosed orthopedic deficits assessed before and after diagnostic analgesia on the straight line and on the lunge.

### Inter-stride variability

In the present study, the high correlation between the proportion of left or right limb asymmetries and the increasing value of HDmin and PDmin (Fig 2) raises important questions when evaluating horses with subtle lameness. For horses with subtle asymmetries, the asymmetries may appear intermittently and would thus not be visible or measurable in every stride. Therefore, both the proportion of lame strides and the magnitude of the asymmetries must be taken into account when evaluating lameness for example before and after diagnostic analgesia. The high inter-stride variability in less asymmetric horses shown here may also contribute to the well documented low inter-rater agreement among veterinarians evaluating subtle lameness. High inter-stride variability may also explain how easily expectation bias can influence the visual assessment when ‘hoping for’ either lame or sound strides after a block or treatment [6]. Peham et al. [25] showed that inter-stride variability of stride length decreased with increasing lameness but the number of left or right lame strides was not studied. It could be possible that high inter-stride variability is a sign of a non-lame horse. A pain free horse may ‘not care’ how it moves in each stride and hence some strides may be more symmetrical than others. In contrast to this, a lame horse in pain could have developed a preferred way of moving that minimises pain, and as a consequence reduces inter-stride variability.

In this study, proportions of left/right asymmetries were close to 50/50%. Therefore, reasons such as rider handedness, or horse laterality or the fact that horses are often handled from the left side are unlikely explanations of the results.

### Limitations of the study

No clinical examination was performed on the horses and the underlying causes of the motion asymmetries are not known. The present study used a convenience sample of horses, stabled within reasonable proximity to the authors. It is possible that some owners offered their horse(s) for participation in the study because they suspected some locomotor problems in their horses, resulting in some ‘inclusion’ bias. However, all horses had been in training for at least 6 months previous to the study without referral to a veterinarian. Some other relations between motion symmetry, performance level, age, withers height and discipline may not have been detected due to a small sample size.

## Conclusions

A major proportion of horses in regular training show motion asymmetries of similar magnitude to asymmetries found in horses examined and treated for lameness. It is not known to what extent these asymmetries are caused by pain and hence may present a potential welfare problem or may be related to poor performance. Further studies are needed to investigate this. It may be a welfare problem, if indeed a proportion of such asymmetries were not painful but caused by inherent natural laterality or by poor/asymmetric training. Increased knowledge about the relationship between pain and movement asymmetry is needed to avoid excessive lameness investigations and unnecessary treatment or euthanasia. One of the most important questions to be addressed before biomechanical objective asymmetry scores can be translated into pathology, is to which extent pain, dysfunction or orthopedic abnormality and motion asymmetry are synonymous conditions. The high inter-stride variability seen in horses presenting mild movement asymmetries may contribute to the well documented low inter-rater agreement among veterinarians evaluating subtle lameness and may also explain how expectation bias can influence visual assessment when expecting either lame or sound strides after a diagnostic block or treatment. To further understand any causal relationship between disciplines, performance level and motion symmetry longitudinal studies are needed.

## Supporting information

S1 Table Least square means of univariable fixed effects from mixed models. Univariable fixed effects with owner as a random effect for two dependent variables (PDmin and PDmax) for variables with group-level p-values <0.05. BT- back-transformed estimate and standard error (SE). NS- non significant pairwise comparison.
(XLSX)

S1 Data Data set used for the data analysis. Excel sheet containing description of variables used for the analysis.
Sheet ‘reduced_content_rowsred’ contains descriptions of the data and explanations of what the variable names mean. Sheet ‘data_rowsred’ contains the data.
(XLSX)
